# Biochemical remission, diagnostic delays, and comorbidities of acromegaly in China: a large single-centre retrospective study

**DOI:** 10.3389/fendo.2025.1526625

**Published:** 2025-02-24

**Authors:** Xue Bai, Lian Duan, Shengmin Yang, Tingyu Wang, Yong Yao, Meng Zhang, Jingya Zhou, Shengnan Cui, Cheng Pang, Yi Wang, Huijuan Zhu

**Affiliations:** ^1^ Department of Medical Records, Peking Union Medical College Hospital, Chinese Academy of Medical Sciences and Peking Union Medical College, Beijing, China; ^2^ Collaborating Center for the WHO Family of International Classifications in China, Peking Union Medical College Hospital, Chinese Academy of Medical Sciences and Peking Union Medical College, Beijing, China; ^3^ Key Laboratory of Endocrinology of National Health Commission, Department of Endocrinology, State Key Laboratory of Complex Severe and Rare Diseases, Peking Union Medical College Hospital, Chinese Academy of Medical Science and Peking Union Medical College, Beijing, China; ^4^ Department of Neurosurgery, Peking Union Medical College Hospital, Chinese Academy of Medical Sciences and Peking Union Medical College, Beijing, China; ^5^ China Pituitary Disease Registry Center, Peking Union Medical College Hospital, Chinese Academy of Medical Sciences and Peking Union Medical College, Beijing, China

**Keywords:** acromegaly, biochemical remission, comorbidity, diagnostic delays, retrospective study

## Abstract

**Introduction:**

Long-term biochemical nonremission and long-delayed diagnosis can increase the incidence of comorbidities of acromegaly and seriously affect patients’ quality of life. To identify predictors of biochemical remission and quantify the relationship between delayed diagnosis and comorbidities, we performed a retrospective study of a large, single-centre cohort.

**Methods:**

This retrospective cohort included 1692 hospitalised patients with acromegaly seen in a single referral centre between 2012 and 2020. To account for the longitudinal data structure, generalised estimating equation (GEE) regression models were established to further evaluate the factors associated with biochemical remission.

**Results:**

Overall, 1692 inpatients (55.4% females, mean age at diagnosis: 40.1 ± 12.2 years, mean age at onset: 34.4 ± 11.71 years, median diagnostic delay: 4.4 years) were included. A total of 86.8% (1306/1504) had macroadenomas, and 34.1% (486/1424) had invasive tumours. According to the international diagnostic criteria, the 5-year biochemical remission rate of this cohort was 26.4%, while the Chinese criterion was 41.4%. According to the GEE model, invasion and large adenoma influence biochemical nonremission. After age 50, comorbidities such as hypertension and hyperlipidaemia were considerably more common in females than in males. The proportion of patients with comorbidities among those with a delayed diagnosis ≥4 years was greater than among those with a delayed diagnosis <4 years (54.9 vs. 47.9%, P=0.004).

**Conclusion:**

The older the age at diagnosis and the longer the delay in diagnosis, the greater the incidence of comorbidities, especially in elderly females. Appropriate treatment of acromegaly should be started early to achieve biochemical control.

## Introduction

Acromegaly is a rare, chronic, progressive endocrine metabolic disease, and 98% of cases are caused by a pituitary growth hormone (GH)-secreting adenoma (1).

The high concentrations of GH and insulin-like growth factor-1 (IGF-1) in the serum of patients with this rare endocrine disease affect their physiology and metabolism and cause a series of symptoms (2) that seriously affect their health and quality of life (3, 4). Patients are usually not diagnosed until long after the onset of the disease, and most are diagnosed only when they seek medical care for comorbidities. A delay in diagnosis (5) significantly increases the incidence of comorbidities and the difficulty of treatment.

Surgery, drug therapy, and radiotherapy are the main treatments for acromegaly. Treatment of acromegaly aims to normalise the levels of GH and IGF-1, alleviate comorbidities, and improve the prognosis and quality of life of patients (6, 7). A Bulgarian national survey of 191 patients revealed that the biochemical remission rate of acromegaly patients within one year was 84.3% (8). In a meta-analysis of 394 participants, the overall achieved control rates were 56% for mean GH normalisation and 55% for IGF-1 normalisation (9).

Although diagnostic and therapeutic methods have advanced, relatively few clinical data exist concerning long-term biochemical remission and chronic comorbidities in patients with acromegaly in China. Using real data from Peking Union Medical College Hospital (PUMCH) inpatients, this study analysed the clinical characteristics, comorbidities, short-term and long-term biochemical remission, and factors influencing acromegaly to understand the biochemical control rate and comorbidities of acromegaly patients in China and provide a detailed reference for their treatment.

## Methods

### Patients

This was a retrospective observational study. All patients diagnosed with acromegaly underwent routine outpatient follow-up. Because we had complete data for the hospitalised acromegaly patients, we took them as the research subjects and analysed their outpatient follow-up data.

**Figure 1:**
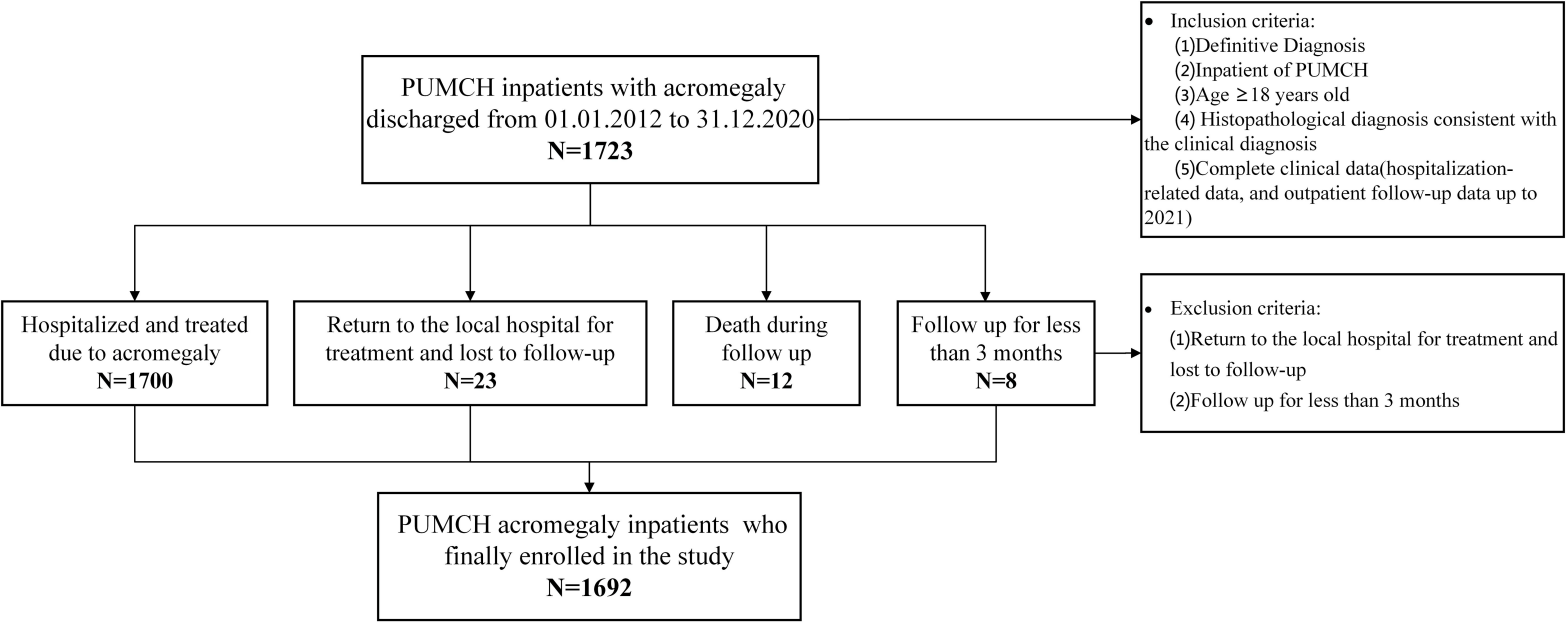
Flow chart of the acromegaly patient selection process in this study.

The inclusion criteria for this study were as follows (10) (1): patients with an established acromegaly diagnosis who were discharged from January 2012 to December 2020; (2) hospitalised patients aged 18 years old or older; (3) histopathological diagnosis consistent with the diagnosis of acromegaly in surgical patients; and (4) complete clinical data in the PUMCH record system. The complete clinical data of patients at PUMCH included hospitalisation-related data and outpatient follow-up data through 2021. All medical data were collected from electronic medical records. First, 1735 patients were selected. After carefully reading the medical records, 23 patients were diagnosed with acromegaly at PUMCH, but they were asked to return to their local hospital for treatment and were thus lost to follow-up; 12 patients died during the follow-up, and 8 patients were followed up for less than 3 months. Ultimately, a total of 1692 patients were included in the study (shown in **Figure 1**).

The study was reviewed and approved by the Ethics Committee of Peking Union Medical College Hospital (approval number K3584).

### Study design

The first-line treatment for newly diagnosed patients in China is surgery. Patients with surgical contraindications or unwillingness to undergo surgery used medication. The main drugs used were octreotide LAR and lanreotide SR injection or dopamine receptor agonist, such as cabergoline or bromocriptine.

To determine which factors influenced the patients’ biochemical remission and reduce the changes in biochemical indicators caused by the continuous addition of new treatment schemes during the treatment process, the starting point of follow-up was determined by combining the patients’ outpatient and inpatient treatment times and the times when treatment was received outside the hospital in the electronic medical records: (1) in the patients who underwent surgery, only the last operation time was taken as the starting point of follow-up; (2) if the patient only received surgery and radiotherapy (no drug treatment), radiotherapy was the starting point of follow-up; and (3) if the patient received surgery and drug treatment, radiotherapy and drug treatment, or all three, the starting time of medication was taken as the starting point of follow-up.

The following concepts were defined in the research: (1) onset time was defined as the time of first symptom or sign onset; (2) diagnostic delay was defined as the time between onset and diagnosis; (3) disease duration was defined as the time from onset to last hospitalisation; (4) the time of disease control was defined as the time of biochemical remission; and (5) active disease duration was defined as the time between onset time and the time of disease control.

### Biochemical evaluation

In order to evaluate biochemical remission, GH and IGF-1 were measured via a fully automated 2-site, solid-phase, chemiluminescent enzyme immunometric assay (Immulite2000; Siemens Healthcare Diagnostics, GH calibrated against the recommended IS 98/574). For IGF-1, the absolute measured values were encoded along with the upper limit of normal for age and sex. The results are expressed as multiples of the upper limit of the normal value (ULN).

Biochemical remission was evaluated based on Endocrine Society and Chinese guidelines. In accordance with the Endocrine Society guidelines (11, 12), the criteria for biochemical remission were as follows: (1) a random GH or OGTT-GH nadir value < 0.4 ng/m and (2) age-adjusted IGF-1 to less than 1.0 ULN. In accordance with Chinese guidelines (10), the criteria for biochemical remission were as follows: (1) a random GH or OGTT-GH nadir value < 1.0 ng/m and (2) a decrease in serum IGF-1 to less than 1.0 ULN (upper limit of the normal value).

### Imaging evaluation

Radiological data for the maximal tumour diameter were used to calculate the proportion of patients with microadenomas (<10 mm) and macroadenomas (≥10 mm) on MRI scans at diagnosis. Tumour invasiveness was judged according to the Knosp classification system: Grade 3 and Grade 4 lesions were considered invasive.

### Predictor variables

Predictor variables were evaluated as possible predictors of remission. Patient factors included sex and age of onset. Clinical factors included diagnostic delay, first surgical procedure and treatment plans. Radiological factors included tumour invasion and the maximum tumour diameter. The biochemical factors included the baseline GH and IGF-1 levels, the GH and IGF-1 levels measured at different time points, and the test time.

### Comorbidity evaluation

The comorbidity data were collected based on diagnoses in electronic medical records at the outpatient or inpatient stage. Hypertension was defined as blood pressure ≥140/90 mmHg or a history of hypertension (previously confirmed hypertension or the administration of antihypertensive medication before blood pressure measurement) (13, 14). Abnormal glucose metabolism, including diabetes and impaired glucose tolerance, was defined as a history of abnormal glucose metabolism or blood glucose meeting diagnostic criteria in the OGTT, according to the 1999 World Health Organization criteria (15, 16). Hyperlipidaemia was defined as a history of hyperlipidaemia, hypertriglyceridaemia (triglyceride ≥ 1.7 mmol/L), or hypercholesterolaemia (total cholesterol ≥ 5.2 mmol/L or LDL-cholesterol ≥ 3.4 mmol/L) (17, 18). OSAHS was defined as a history of OSAHS or meeting polysomnography (PSG) diagnostic criteria (adults: AHI ≥5/h) (19, 20).

Anterior hypopituitarism was defined as the loss of one or more anterior pituitary axes (21–24), leading to secondary adrenal insufficiency (fasting serum cortisol in the morning <3.0 μg/dL with a low or normal ACTH level), secondary hypothyroidism (free thyroxine less than 0.81 ng/dL, with thyroid-stimulating hormone low or normal levels) and hypogonadotropic hypogonadism. Hypogonadotropic hypogonadism has different diagnostic criteria for males and females. Hypogonadotropic hypogonadism in males was defined as a serum testosterone concentration less than 3.0 ng/mL and low or normal follicle-stimulating hormone and luteinising hormone levels (<10 IU/L). Hypogonadotropic hypogonadism in females was defined as (1) lower levels of luteinising hormone (<25 IU/L) and follicle-stimulating hormone (<40 IU/L) in postmenopausal patients and (2) menstrual disorders and a low oestradiol level (<30 pg/mL) accompanied by low or low-normal luteinising hormone and follicle-stimulating hormone (<10 IU/L) in premenopausal patients.

### Statistical analyses

Categorical variables are presented as numbers and proportions. Continuous variables are presented as the mean ± standard deviation or median plus interquartile range, according to the data distribution tested by the Kolmogorov–Smirnov test. To account for the longitudinal data structure, generalised estimating equation (GEE) regression models were built to further evaluate the factors associated with biochemical remission at three months, six months, 12 months, 2 years, 3 years, and 5 years after treatment. A first-order autoregressive AR-1 was selected as the working correlation structure, which allowed the correlations of measurements taken farther apart to be less than those taken closer to one another. The biochemical remission model included time and factor variables that may clinically affect biochemical remission. The estimates are presented as P values and odds ratios with 95% confidence intervals. Data management and analysis were performed with R version 4.0.2 (tidyverse, compareGroups, geepack, sjPlot, ggsankey). Graphs were generated with Prism 9.3.0.

## Results

### Clinical and demographic characteristics

Among the 1692 inpatients admitted to PUMCH between January 2012 and December 2020, 937 (55.4%) were female and 755 (44.6%) were male. Among these patients, 1410 (83.3%) came from outside Beijing, 1597 (94.4%) were of Han nationality, and 1410 (83.3%) were first diagnosed in the neurosurgery department. The mean age was 40.1 ± 12.2 years at diagnosis and 34.4 ± 11.7 years at onset. The mean ages at diagnosis for males and females were 38.1 ± 11.3 years and 41.7 ± 12.6 years, respectively. The median disease duration was ten years. Among all patients, the median diagnostic delay was 52.5 months (4.4 years), and 53.5% of the acromegaly patients had a diagnostic delay of more than four years. The median diagnostic delays for males and females were 61.0 months (5.1 years) and 44.0 months (3.7 years), respectively.

A total of 27.1% of the patients (458) had more than three comorbidities (counting all comorbidities from onset to last hospitalisation). A total of 97.1% (1643/1692) of the patients underwent surgery, and 68.4% (1158/1692) of the patients received only surgical treatment. A total of 86.8% (1306/1504) of the patients had macroadenomas, and 34.1% (486/1424) had invasive tumours, which were more common in females than in males (64.2% vs. 35.8%, P<0.001). See **Table 1**.

**Table 1:** Patients characteristics of acromegaly in PUMCH.

Variable	N	Value
**All patients**	1692	
**Sex, n (%)**	1692	
Male		755 (44.6)
Female		937 (55.4)
**Age at diagnosis, years**	1692	40.1± 12.2
**Age of onset, years**	1690	34.4 ± 11.7
**Disease duration, years**	1690	10.0 (6.0, 13.0)
**Diagnostic delay, months**	1690	52.5 (24, 97.25)
**Maximum tumor diameter (mm)**	1504	16.0 (12.0, 22.0)
**Baseline random GH, ng/mL**	652	10.0 (4.3, 22.5)
**Baseline OGTT nadir GH, ng/mL**	904	8.4 (3.7, 18.5)
**Baseline IGF-1, ULN**	1342	3.0 (2.3, 3.6)
**Ethnicity, n (%)**	1692	
Han nationality		1597 (94.4)
Minority nationality		95 (5.6)
**Region, n (%)**	1692	
Beijing		282 (16.7)
Other cities		1410 (83.3)
**First visit department, n (%)**	1692	
Neurosurgery		1410 (83.3)
Endocrinology		263 (15.5)
Others		19 (1.1)
**Adenoma sizes, n (%)**	1504	
Micro-adenoma (<10 mm)		198 (13.2)
Macro-adenoma (≥10 mm)		1306 (86.8)
**Invasion, n (%)**	1424	
Yes		486 (34.1)
No		938 (65.9)
**Diagnostic delay, n (%)**	1690	
<4 year		785 (46.4)
≥4 year		905 (53.6)
**Treatments patterns, n (%)**	1692	
Surgeries		1158 (68.4)
Medical		37 (2.2)
Radiotherapy		2 (0.1)
Surgeries and Medical		250 (14.8)
Medical and Radiotherapy		10 (0.6)
Surgeries and Radiotherapy		95 (5.6)
Surgeries, Medical and Radiotherapy		140 (8.3)
**First treated with surgeries, n (%)**	1500	
Microscopic approach		1110 (74.0)
Endoscopic approach		377 (25.1)
Open operation		13 (0.9)
**Number of comorbidities, n (%)**	1692	
<3		1234 (72.9)
≥3		458 (27.1)

Values are presented as the mean ± standard deviation, number (%), or median (interquartile range), according to the distribution of data. Bold values are primary classifications that describe the basic characteristics of patients, followed by the second level classification under this category.

**Figure 2:**
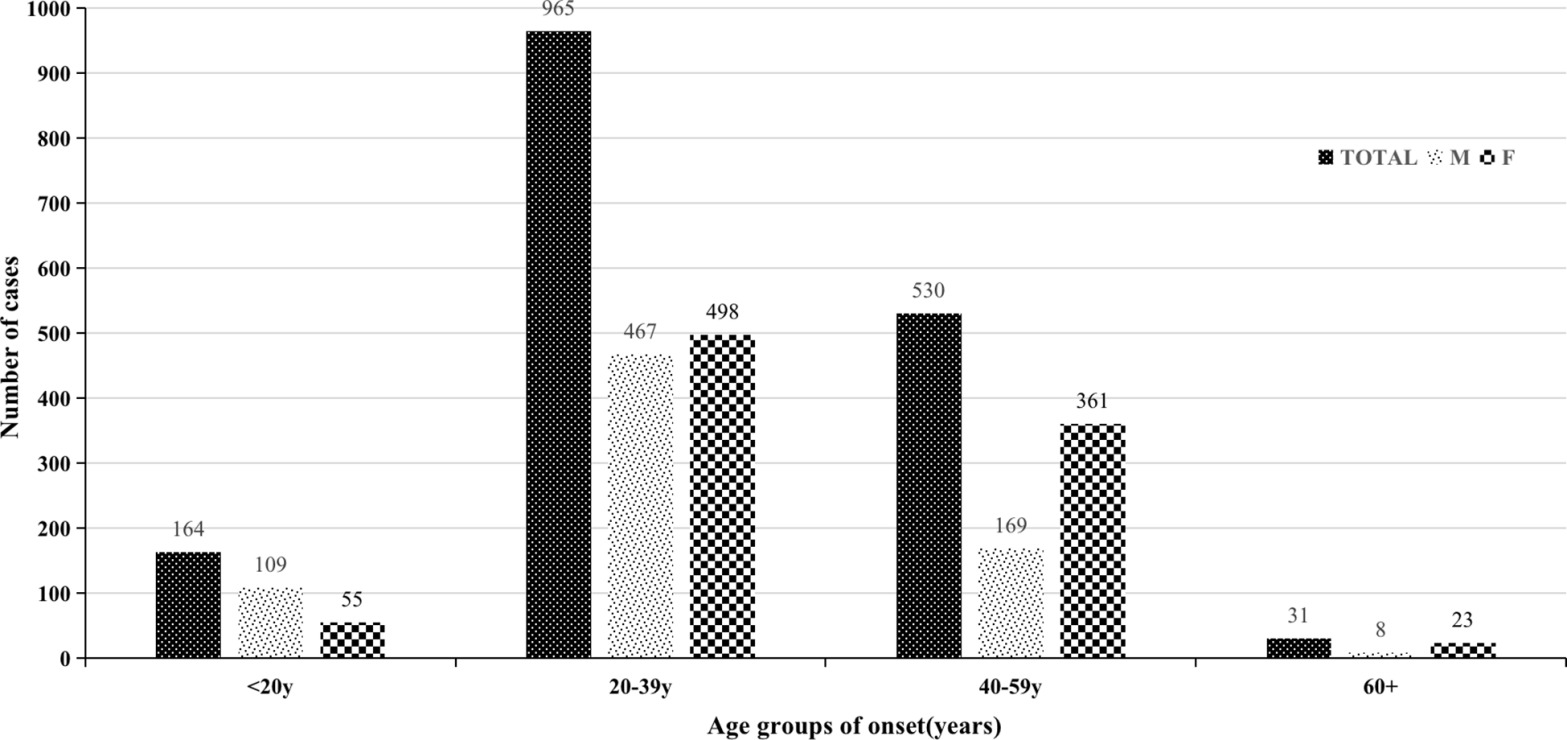
Total number and four differences in different age groups at onset.

To understand the incidence of acromegaly, the age of onset was divided into four twenty-year intervals. As shown in **Figure 2**, acromegaly was more common among males than females aged less than 20 years (M/F=2.0). Most of the patients with acromegaly were aged 20–39 years (965/1692, 57.0%), and the ratio of males to females was balanced (1.0/1.1). In the more than 40-year-old groups, there were significantly more female patients than male patients (F/M: overall: 384/177; 40–59 years: 2.1/1; 60+ years: 2.9/1).

### Treatment outcomes and predictors of biochemical remission

**Table 2** and the **Supplementary Table** show the number and proportion of acromegaly patients in biochemical remission after 7 follow-up periods, including 1–3 months, 3–6 months, 6–12 months, 1–2 years, 2–3 years, 3–5 years and more than 5 years. The median follow-up time among all patients from the starting point of follow-up to the time of the last test was 2.0 (0.3, 4.1) years.

**Table 2:** Number and proportion of biochemical remission in acromegaly patients in seven different follow-up time groups according to international diagnostic criteria.

Characteristic	Less than 3 months (n=317)	≥3 and <6 months (n=670)	≥6 and ≤12 months (n=381)	>1 and ≤2 years (n=555)	>2 and ≤3 years (n=350)	>3 and ≤5 years (n=410)	More than 5 years (n=220)
**Sum of remission, n (%)**	44 (13.9)	159 (23.7)	61 (16.0)	125 (22.5)	92 (26.3)	101 (24.6)	58 (26.4)
Sex, n (%)							
Male	23 (16.8)	78 (26.4)	22 (15.3)	61 (27.2)	42 (27.5)	47 (31.1)	28 (31.5)
Female	21 (11.7)	81 (21.7)	39 (16.5)	64 (19.3)	50 (25.4)	54 (20.8)	30 (22.9)
Age of onset, n (%)							
<20years	2 (7.4)	17 (27.0)	7 (19.4)	8 (14.3)	12 (28.6)	13 (28.3)	10 (32.3)
20-39years	23 (11.6)	76 (20.1)	24 (10.1)	66 (20.6)	47 (22.2)	51 (20.3)	35 (26.9)
40-59years	19 (22.4)	61 (28.9)	27 (27.0)	47 (28.1)	31 (34.4)	36 (33.3)	13 (23.2)
≥60years	0 (0.0)	5 (29.4)	3 (42.9)	4 (36.4)	1 (20.0)	1 (20.0)	0 (0.0)
Treatments patterns, n (%)							
Surgeries	34 (24.6)	136 (29.9)	49 (26.9)	101 (31.4)	71 (38.0)	75 (34.7)	36 (33.6)
Medical	0 (0.0)	3 (15.8)	0 (0.0)	1 (5.9)	1 (12.5)	0 (0.0)	0 (0.0)
Surgeries and Medical	8 (9.0)	18 (16.4)	11 (12.2)	14 (13.6)	19 (25.0)	16 (21.1)	16 (33.3)
Surgeries and Radiotherapy	1 (9.1)	2 (6.5)	1 (4.3)	4 (14.8)	1 (5.6)	2 (8.0)	0 (0.0)
Medical and Radiotherapy	0 (0.0)	0 (0.0)	0 (0.0)	1 (20.0)	0 (0.0)	0 (0.0)	1 (25.0)
Surgeries, Medical and Radiotherapy	1 (1.8)	0 (0.0)	0 (0.0)	4 (5.1)	0 (0.0)	8 (11.1)	5 (12.2)
First treated with surgeries, n (%)							
Microscopic approach	24 (16.9)	105 (25.5)	35 (17.3)	92 (26.9)	64 (28.8)	76 (30.6)	45 (30.2)
Endoscopic approach	17 (17.3)	48 (27.0)	24 (20.7)	30 (22.9)	27 (36.0)	22 (25.9)	10 (32.3)
Open operation	0 (0.0)	1 (20.0)	2 (28.6)	0 (0.0)	0 (0.0)	1 (25.0)	0 (0.0)
Starting time of follow-up, n (%)							
2012~2015	12 (14.8)	47 (25.1)	26 (21.3)	40 (20.4)	36 (24.2)	46 (25.7)	44 (26.8)
2016~2019	29 (14.3)	101 (23.0)	29 (12.5)	78 (24.6)	56 (28.6)	55 (25.0)	10 (34.5)
2020~2021	3 (9.1)	11 (25.0)	6 (22.2)	6 (16.2)	–	–	–
Adenoma sizes, n (%)							
Micro-adenoma (<10 mm)	13 (37.1)	25 (31.6)	13 (35.1)	22 (33.8)	13 (35.1)	12 (27.3)	13 (48.1)
Macro-adenoma (≥10 mm)	28 (12.7)	130 (25.0)	48 (16.6)	101 (24.3)	79 (30.3)	86 (29.0)	42 (27.6)
Invasion, n (%)							
Yes	7 (7.9)	18 (10.2)	11 (9.7)	24 (15.6)	17 (19.1)	21 (17.4)	14 (20.9)
No	32 (23.9)	135 (34.4)	50 (27.2)	95 (32.3)	70 (38.9)	73 (38.2)	40 (39.2)
Diagnostic delay, n (%)							
<4 year	14 (9.5)	69 (22.5)	25 (12.9)	45 (16.5)	27 (16.7)	53 (24.1)	22 (22.4)
≥4 year	30 (17.8)	90 (24.8)	36 (19.3)	80 (28.3)	64 (34.2)	48 (25.3)	36 (29.5)

According to the Endocrine Society guidelines, among patients followed up for more than five years, 29.9% achieved GH remission, 57.1% achieved IGF-1 remission, and 26.4% achieved biochemical remission. Biochemical remission was observed in 13.9% of patients at 0–3 months, which increased to 23.7% at 3–6 months, decreased rapidly to 16.0% at 6–12 months, and again increased to 26.3% at 2–3 years. Finally, 26.4% of patients experienced biochemical remission after 5 years. As the follow-up progressed, the proportions of patients who experienced GH remission only (P<0.001), IGF-1 remission only (P<0.001), and biochemical remission (P<0.001) gradually increased. The GEE model (**Table 3**) was used to analyse the factors influencing biochemical remission in acromegaly patients. According to the biochemical remission model, invasion (vs. noninvasive) (OR, 2.26; 95% CI, 1.66–3.10; P<0.001) and maximal tumour diameter (OR, 1.40; 95% CI, 1.17–1.68; P<0.001) were risk factors for nonremission. Age of onset between 40 and 59 years (vs. 20–30 years) (OR, 0.63; 95% CI, 0.48–0.84; P=0.002) was a protective factor for nonremission.

**Table 3:** GEE model analysis for influencing factors of biochemical remission in acromegaly patients with different treatment pattern.

Characteristic	International diagnostic criteria									Chinese diagnostic criteria								
	All patients			Only surgical treatment			Other treatments			All patients			Only surgical treatment			Other treatments		
	OR	95%CI	P	OR	95%CI	P	OR	95%CI	P	OR	95%CI	P	OR	95%CI	P	OR	95%CI	P
Age of onset, years																		
20-39year	Ref			Ref			Ref			Ref			Ref			Ref		
<20year	0.94	0.60-1.45	0.771	0.69	0.40-1.20	0.190	0.93	0.60-1.45	0.757	0.96	0.63-1.45	0.848	0.74	0.43-1.27	0.269	0.93	0.61-1.41	0.730
40-59year	0.63	0.48-0.84	0.002	0.69	0.51-0.95	0.024	0.68	0.51-0.90	0.008	0.62	0.48-0.80	<0.001	0.65	0.48-0.87	0.004	0.65	0.50-0.85	0.002
60+	0.66	0.25-1.69	0.382	0.77	0.28-2.09	0.607	0.70	0.27-1.83	0.467	0.50	0.21-1.18	0.115	0.55	0.23-1.34	0.190	0.54	0.23-1.28	0.163
Maximal tumor diameter	1.40	1.17-1.68	<0.001	1.45	1.16-1.82	0.001	1.41	1.17-1.71	<0.001	1.27	1.08-1.49	0.004	1.26	1.03-1.54	0.026	1.26	1.07-1.49	0.007
Diagnostic delay, month																		
<2.0year	Ref			Ref			Ref			Ref			Ref			Ref		
2.0-4.4year	1.05	0.72-1.53	0.818	1.28	0.83-1.98	0.262	1.04	0.71-1.52	0.860	1.10	0.78-1.53	0.597	1.22	0.83-1.81	0.312	1.09	0.77-1.53	0.625
4.4-8.1year	0.72	0.49-1.05	0.084	0.86	0.56-1.33	0.504	0.75	0.51-1.10	0.139	0.89	0.63-1.27	0.529	1.04	0.70-1.55	0.853	0.94	0.66-1.33	0.724
8.1+	0.96	0.66-1.39	0.834	1.18	0.77-1.80	0.444	0.99	0.68-1.44	0.948	1.03	0.74-1.45	0.847	1.18	0.80-1.74	0.413	1.07	0.76-1.51	0.681
Sex																		
Female	Ref			Ref			Ref			Ref			Ref			Ref		
Male	0.84	0.65-1.09	0.189	0.90	00.67-1.20	0.470	0.82	0.63-1.06	0.125	1.09	0.86-1.38	0.494	1.06	0.80-1.39	0.689	1.07	0.84-1.35	0.601
Surgeries																		
No	Ref			–	–	–	Ref			Ref			–	–	–	Ref		
Yes	0.14	0.02-1.24	0.077	–	–	–	0.49	0.36-0.68	<0.001	0.09	0.01-0.56	0.010	–	–	–	0.55	0.42-0.73	<0.001
First treated with surgeries																		
Open operation	–	–	–	Ref			Ref			–	–	–	Ref			Ref		
Endoscopic approach	–	–	–	5.22	0.38-70.83	0.214	4.55	0.92-22.42	0.062	–	–	–	2.80	0.17-46.32	0.471	2.43	0.49-12.08	0.278
Microscopic approach	–	–	–	4.92	0.36-66.54	0.230	4.41	0.91-21.47	0.066	–	–	–	2.89	0.18-47.48	0.458	2.72	0.55-13.38	0.220
Invasion																		
No	Ref			Ref			Ref			Ref			Ref			Ref		
Yes	2.26	1.66-3.10	<0.001	1.95	1.34-2.81	<0.001	2.13	1.55-2.93	<0.001	2.04	1.54-2.70	<0.001	1.70	1.23-2.35	0.001	1.93	1.46-2.54	<0.001

According to the Chinese diagnostic criteria, among patients followed up for more than five years, 52.6% achieved GH remission, 57.1% achieved IGF-1 remission, and 41.4% achieved biochemical remission. Biochemical remission was observed in 19.8% of patients at 0–3 months, which increased to 30.4% at 3–6 months, decreased rapidly to 22.8% at 6–12 months, and again increased to 42.0% at 2–3 years. Finally, 41.4% of patients experienced biochemical remission after 5 years. (**Supplementary Tables 2**, **3**).

### Different treatment patterns as predictors of remission

All patients were grouped by treatment method to judge the effects of the different treatment methods on biochemical remission. The GEE model (**Table 3**) was applied to different subgroups to identify their own influencing factors.

According to the Endocrine Society guidelines, in the surgical treatment subgroup, as the follow-up time increased, the proportion of patients who achieved biochemical remission increased (P<0.001). Invasion (vs. noninvasive) (OR, 1.95; 95% CI, 1.34–2.81; P<0.001) and maximal tumour diameter (OR, 1.45; 95% CI, 1.16–1.82; P=0.001) were risk factors for biochemical nonremission. Age of onset between 40 and 59 years (vs. 20–30 years) (OR, 0.69; 95% CI, 0.51–0.95; P=0.024) was a protective factor for nonremission. In patients who underwent other treatments (surgery and medical treatment; surgery and radiotherapy; medical treatment and radiotherapy; and surgery, medical treatment and radiotherapy), biochemical remission became more common with longer follow-up. As the follow-up progressed, the proportion of patients who experienced biochemical remission gradually increased (P<0.001). Invasion (OR, 2.13; 95% CI, 1.55–2.93; P<0.001) and maximal tumour diameter (OR, 1.41; 95% CI, 1.17–1.71; P<0.001) were risk factors for nonremission. Age of onset between 40 and 59 years (vs. 20–30 years) (OR, 0.68; 95% CI, 0.51–0.90; P=0.008) and surgical treatment (vs. no surgical treatment) (OR, 0.49; 95% CI, 0.36–0.68; P<0.001) were protective factors for nonremission.

### Comorbidities of acromegaly

Among all 1692 patients, 874 (51.7%) had at least one comorbidities at the time of diagnosis, and the median number of comorbidities was one. The incidence of 5 common comorbidities were as follows: abnormal glucose metabolism (29.8%, n=504), hypertension (28.3%, n=479), hyperlipidaemia (14.0%, n=237), OSAHS (12.6%, n=213), and anterior hypopituitarism (12.6%, n=173).

**Figure 3:**
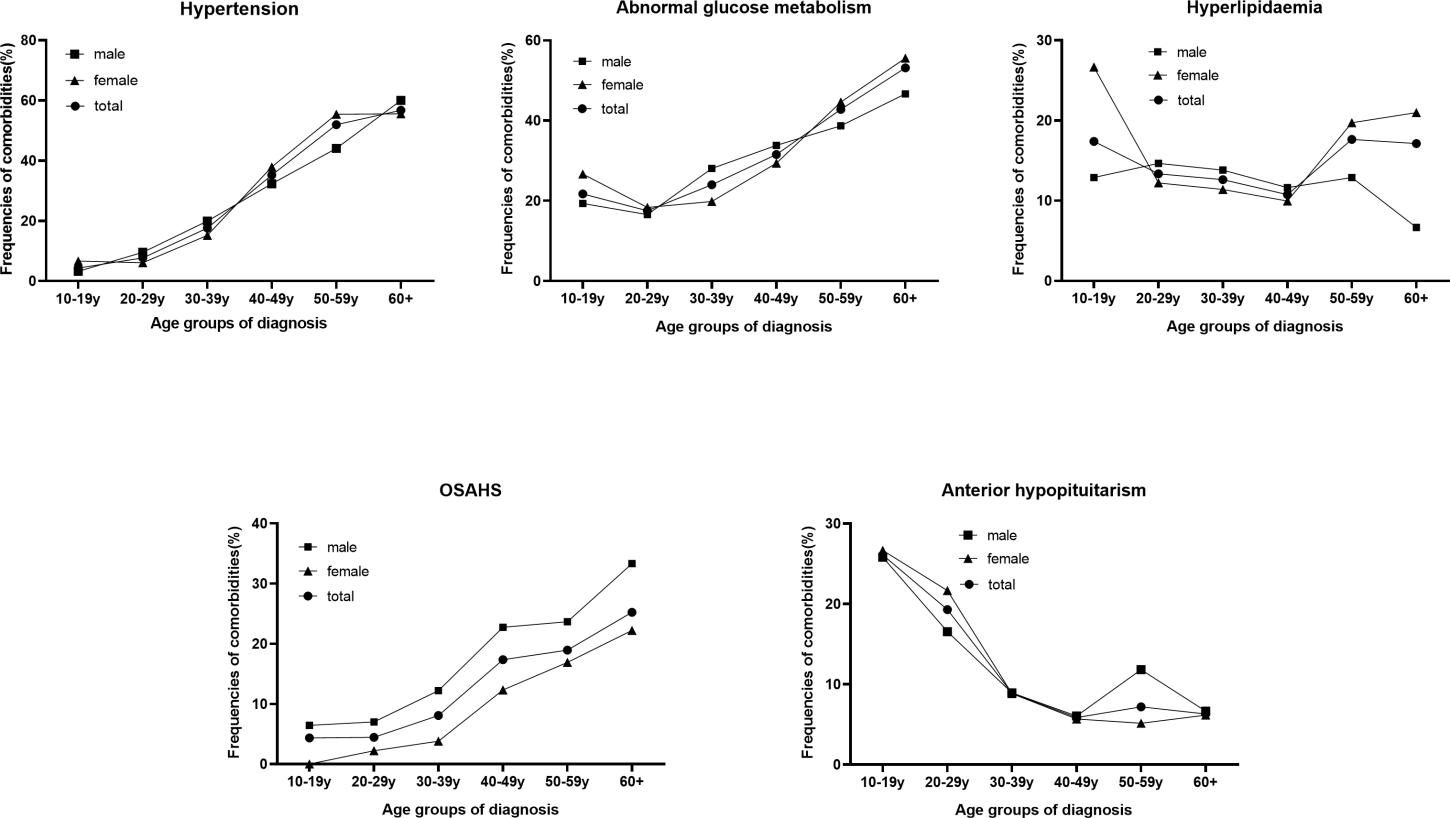
Proportion of comorbidities between males and females in different age groups at diagnosis.

The incidence of 5 comorbidities classified by sex is shown in **Figure 3**. Overall, these comorbidities became more common with older age at diagnosis (from 47.8% at 10-19 y to 78.4% at 60+ y, P<0.001) and was greater in females than in males (53.5 vs. 49.4%, P=0.096). Males younger than 50 years had more comorbidities than females (23.0% vs. 21.5%, P<0.001), while females older than 50 years had more than males (53.2% vs. 19.2%, P<0.001).

Hypertension is one of the most common comorbidities of acromegaly, and its incidence increases with age at diagnosis (from 4.4% at 10-19 y to 56.8% at 60+ y, P<0.001) and is greater in females than in males (31.1 vs. 24.9%, P=0.005). Hypertension was more common in females than males over 40 (48.1 vs. 38.3%, P=0.006). The incidence of abnormal glucose metabolism increased with age at diagnosis (from 21.7% at 10-19 y to 53.2% at 60+ y, P<0.001). The proportion of hyperlipidaemic patients decreased with age at diagnosis but increased after 50 years of age (from 17.4% at 10-19 years to 17.7% at 50+ years, P=0.102). Among patients older than 50 years, females were more likely to have hyperlipidaemia than males were (20.1% vs. 11.4%, P=0.031). The proportion of patients with OSAHS increased rapidly from 4.4% at 10-19 y to 25.2% at 60+ y (P<0.001) and was lower in females than in males (9.9 vs. 15.9%, P<0.001). Among patients younger than 50 years, males were more likely to have OSAHS than females (13.9% vs. 6.1%, P<0.001). The incidence of anterior hypopituitarism decreased rapidly (from 26.1% at 10-19 y to 6.3% at 60 y, P<0.001).

### Impact of delayed diagnosis on comorbidities

**Figure 4:**
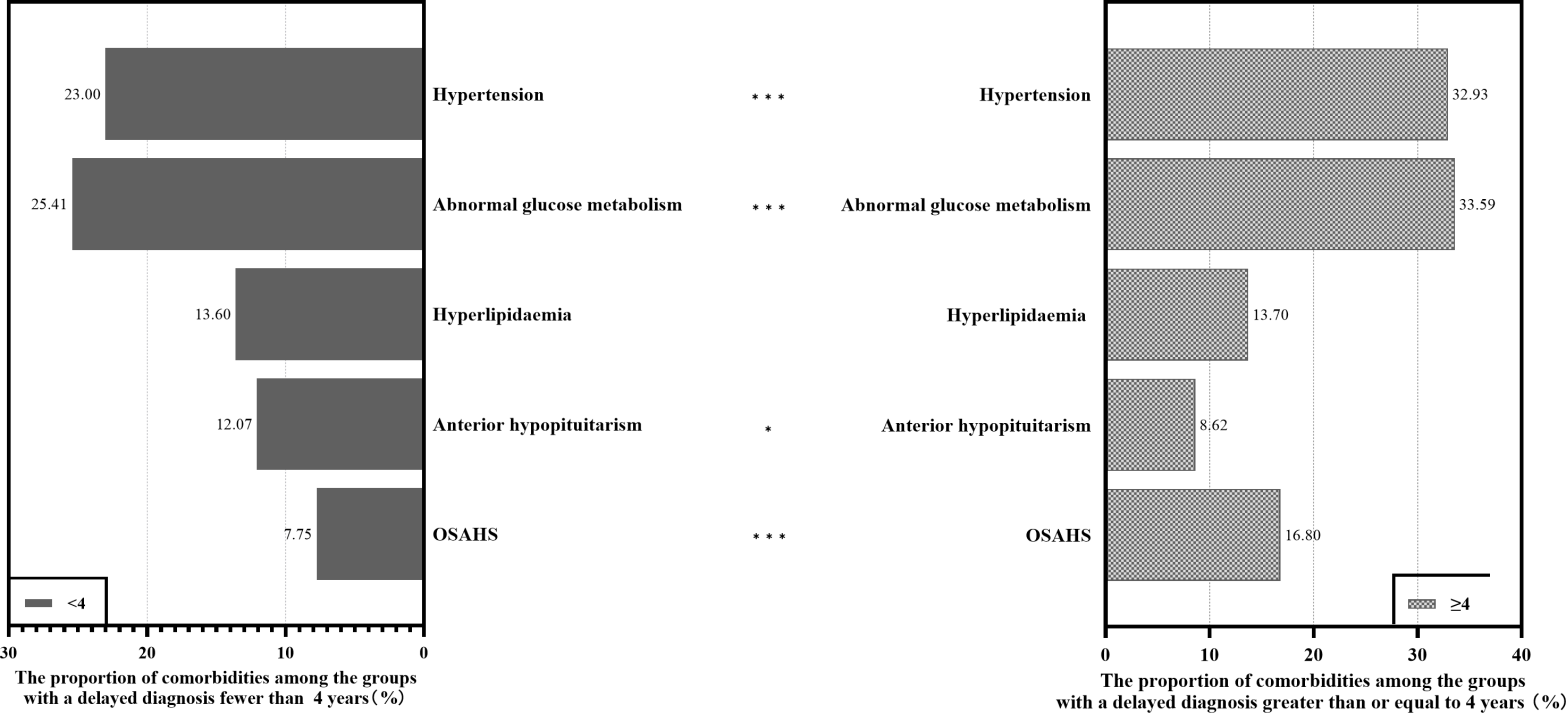
Proportion of comorbidities among the groups with a delayed diagnosis between ≥4 years and ≤4 years. * indicates that the difference in the number of comorbidities is significant (P<0.05). ***indicates that the difference in the number of comorbidities is exceptionally significant (P<0.001).

The comorbidities were divided into two groups around the median number of years since diagnosis. Overall, the patients whose diagnosis was delayed ≥4 years were more likely to have comorbidities than patients with a <4-year delay in diagnosis (54.9 vs. 47.9%, P=0.004). Compared to the group with a delayed diagnosis of <4 years, the group with a ≥4-year delay had significantly higher proportions of hypertension (32.9 vs. 23.0%, P<0.001), abnormal glucose metabolism (33.6 vs. 25.4%, P<0.001), hyperlipidaemia (13.7 vs. 13.6%, P=0.950), and OSAHS (16.8 vs. 7.8%, P<0.001). But in the group with a ≥4-year delay, the proportion of anterior hypopituitarism (8.6 vs. 12.1%, P=0.019) was significantly less. (shown in **Figure 4**).

## Discussion

This study included 1692 patients from a single centre over eight years, including 29.3% of patients who received multimodal treatment. Biochemical data and multiple measurement comparisons were used to observe patient biochemical remission, identify factors influencing biochemical remission, and compare comorbidity rates. This study provides a representative and detailed understanding of acromegaly in China from multiple perspectives.

In our cohort, most patients were female (55.4%), which is consistent with results from other national centres (25–27). Macroadenomas accounted for most cases (86.8%) in this study, but a greater proportion of females (55.8 vs. 44.2%, P=0.039) than males had macroadenomas, which was different from the findings of other studies (28, 29). The average age at onset in this study was 34.4 ± 11.7 years, which is slightly greater than the 33.5 years reported in the EU multicentre study (25). According to guidelines (10), the peak age at the time of acromegaly diagnosis is 40–50 years, whereas the peak age at the time of diagnosis in this study was 30–39 years (**Supplementary Figure 1**). Therefore, we compared the delay in diagnosis between different age groups and found that the older the patient was at the time of diagnosis, the longer the time to diagnosis was (P<0.001). This may be because young patients take the initiative to seek medical attention after symptoms appear to screen for diseases earlier.

The median diagnostic delay time was 4.4 years in the cohort. The median diagnostic delay time for males was 5.1 years, which was longer than the 3 years reported in a cross-sectional study of a British population (30), shorter than the 5.5 years reported in the Danish national cohort study (31), and much shorter than the 8 years reported in the EU multicentre study (25). The average diagnostic delay time for females was 3.7 years, which was longer than the 3 years reported in Denmark (31), shorter than the 6 years reported in a study of the UK community (30) and much shorter than the 10 years reported in a study conducted in the EU (25). We found that although the definition of delayed diagnosis was consistent among several centres, there were great differences in the results. This may be due to differences in medical conditions and health awareness between different regions or to multiple confounding factors, such as retrospective and subjective delayed diagnosis. Therefore, in our centre, the time to diagnosis was shorter in females of childbearing age because such patients are likely to seek medical attention earlier, particularly for menstrual disorders or amenorrhea. However, older patients often ignore comorbidities because hypertension and diabetes are very common in their age group, which leads them to ignore the secondary causes of hypertension and diabetes. The presence of macroadenomas has been correlated with delayed diagnosis (32), and the delayed diagnosis time of macroadenomas is longer than microadenomas in this study (4.7 vs. 2.5 years, P=0.019).

According to international standards, the 5-year biochemical remission rate of the cohort was 26.4%, which was much lower than the 41.4% reported by Chinese standards. One of the reasons for the low remission rate was that international standards have stricter requirements for GH remission than Chinese standards do. Nevertheless, in some medical records, it was only refined to a GH concentration less than 1 ng/m; therefore, the use of these data cannot be used to evaluate whether a patient’s biochemical remission meets international standards, leading to an underestimation of the remission rate. The 5-year biochemical remission rate of 41.4% in our cohort was lower than the pooled remission rate of 55% reported in the 2016 meta-analysis (33) and far lower than the approximate rate of 75% for long-term postoperative biochemical control reported in other relevant studies (34–36). In this cohort, many patients had macroadenomas (86.8%) or invasive tumours (34.1%), which were the main factors leading to a lower long-term remission rate. PUMCH is a treatment centre for complex pituitary diseases in mainland China. The patients who received treatment had relatively complex conditions, and 13.1% (215/1643) of the patients received at least 2 surgical treatments.

The other reason may be that many patients with approximately normal IGF-1 levels refused to receive an OGTT in our centre, which left only random GH (without nadir GH) for the analysis during follow-up. Compared with the rapid decrease in GH after treatment, IGF-1 decreased steadily after treatment (9, 37). These findings indicate that GH is more sensitive for detecting short-term remission, whereas IGF-1 is more accurate for detecting long-term remission. Preoperative clinical and biochemical characteristics could impact the likelihood of biochemical remission (38), so the low remission rate of this study may be related to the high proportion of large and invasive tumours in our cohort. Compared with patients who have already achieved biochemical remission, patients who have not yet achieved remission are more willing to visit the hospital for follow-up, which can lead to selection bias.

Like most studies (38, 39), this study revealed that invasion was the most critical factor influencing the impact of biochemical remission. One previous study (39) reported that premenopausal females had a lower long-term surgical remission rate, but the results of this cohort revealed that there was no significant difference in long-term remission between females and males under multimodal treatment. Compared with the elderly group in a retrospective Korean study, the young group had more difficulty achieving long-term surgical remission (39). In some other studies, patients with large adenomas had more difficulty achieving postoperative remission. Similarly, the results of this cohort also showed that after multimodal treatment, large adenomas made it difficult to achieve biochemical remission. Compared with patients aged 20–39 years(young group), those aged 40–59 years(elderly group) were more likely to achieve long-term remission. With longer follow-up, patients gradually received more treatments, and the biochemical remission rate also increased steadily. Compared with nonsurgical treatment, surgery was beneficial for long-term biochemical remission.

The prevalence of diabetes mellitus in acromegaly patients is 12% to 53% (40). The 25% prevalence rate of diabetes in this cohort is far greater than that of the general population in China (12.8%) (41). In this study, 12.6% of the patients had OSAHS, far lower than the 45-80% reported in other studies (42). This study also revealed that older age and delayed diagnosis significantly affected the incidence of OSAHS (43), and the prevalence in males was greater than that in females (6.9 vs. 3.1%). Most of these comorbidities increased with advancing age, but the prevalence of anterior hypopituitarism decreased significantly with age. One possible reason is that the most common type of anterior hypopituitarism is gonadal axis dysfunction, while young patients tend to pay more attention to gonadal function (such as female menstrual disorders and amenorrhea; decreased male sexual function), while elderly patients may overlook gonadal dysfunction. In general, patients with and without comorbidities had similar GH remission rates (P=0.673) and IGF-1 remission rates (P=0.156). After age 50, comorbidities such as hypertension, abnormal glucose metabolism, dyslipidaemia, and OSASH were far more frequent in females than in males. Is this sexual dimorphism caused by oestrogen/GH resistance? After 50 years of age, there was no difference in GH remission between the sexes (P=0.451).

This study has several limitations. First, although the sample size was large, few patients were included at each follow-up. Although the patients were initially treated with surgery or were seen in internal medicine departments, not all the patients were comprehensively evaluated. The comorbidities diagnosed by internal medicine physicians and surgeons were also inconsistent, which might have caused us to overlook relevant comorbidities, such as anterior hypopituitarism. Second, in the interest of data integrity, this study included only inpatients. However, many patients with acromegaly who have no surgical indications or are unwilling to undergo surgery will only be followed up in the outpatient department rather than be hospitalised, and this result will be biased towards patients with surgical indications. Third, in the analysis of factors influencing patients’ biochemical remission, baseline levels of preoperative GH and IGF-1, pathological Ki67, and other indicators were not included. Finally, this study analysed the relationship between comorbidities in patients with delayed diagnoses and diagnosis age and tried to determine the onset time of patients through external hospital medical records as much as possible. It was still inevitable that delayed diagnosis was retrospective and subjective and may be influenced by multiple confounding factors, leading to biased results.

## Conclusion

The older the patient is at the time of acromegaly diagnosis, the longer the delay in diagnosis. This fact has adverse effects on hormone control and the treatment of comorbidities in acromegaly patients. Invasive and macroadenomas are not conducive to the long-term biochemical remission, which can be achieved with surgical treatment. Appropriate treatment of acromegaly should be started early to achieve biochemical control.

## Data Availability

The raw data supporting the conclusions of this article will be made available by the authors, without undue reservation.
