# Supplementary material for: Biochemical remission, diagnostic delays, and comorbidities of acromegaly in China: a large single-centre retrospective study
Source: Front Endocrinol (Lausanne). 2025 Feb 24;16:1526625. doi: 10.3389/fendo.2025.1526625 (PMC11891042; doi:10.3389/fendo.2025.1526625)
Supplement: Supplementary file 1 [file DataSheet1.docx]

**Supplementary table1**

**Number and proportion of GH remission, IGF-1 remission in acromegaly patients in seven different follow-up time groups according to international diagnostic criteria**

| **Characteristic** | **Remission of two models per biochemical examination period** | | | | | | | | | | | | | |
| --- | --- | --- | --- | --- | --- | --- | --- | --- | --- | --- | --- | --- | --- | --- |
|  | **Less than 3 months** | | **≥3 and <6 months** | | **≥6 and ≤12 months** | | **>1 and ≤2 years** | | **>2 and ≤3 years** | | **>3 and ≤5 years** | | **More than 5 years** | |
|  | **GH**  **(n=332)** | **IGF-1**  **(n=344)** | **GH**  **(n=708)** | **IGF-1 (n=698)** | **GH**  **(n=397)** | **IGF-1 (n=406)** | **GH**  **(n=576)** | **IGF-1**  **(n=611)** | **GH**  **(n=387)** | **IGF-1**  **(n=406)** | **GH**  **(n=431)** | **IGF-1**  **(n=447)** | **GH**  **(n=268)** | **IGF-1**  **(n=245)** |
| **Sum of remission, n (%)** | 73(22.0) | 103(29.9) | 279(39.4) | 253(36.2) | 99(24.9) | 130(32.0) | 194(33.7) | 265(43.4) | 119(30.7) | 222(54.7) | 121(28.1) | 235(52.6) | 80(29.9) | 140(57.1) |
| **Sex, n (%)** |  |  |  |  |  |  |  |  |  |  |  |  |  |  |
| Male | 39(27.9) | 40(26.7) | 147(47.4) | 111(35.5) | 40(26.1) | 41(26.3) | 102(43.4) | 116(44.8) | 57(35.0) | 92(51.7) | 56(35.0) | 83(49.7) | 38(33.0) | 56(57.1) |
| Female | 34(17.7) | 63(32.5) | 132(33.2) | 142(36.9) | 59(24.2) | 89(35.6) | 92(27.0) | 149(42.3) | 62(27.7) | 130(57.0) | 65(24.0) | 152(54.3) | 42(27.5) | 84(57.1) |
| **Age of onset, n (%)** |  |  |  |  |  |  |  |  |  |  |  |  |  |  |
| <20years | 5(17.2) | 6(19.4) | 25(37.9) | 26(38.2) | 8(21.1) | 13(34.2) | 16(28.1) | 26(42.6) | 15(33.3) | 27(56.3) | 16(33.3) | 28(56.0) | 13(32.5) | 18(56.3) |
| 20-39years | 39(18.9) | 58(26.9) | 141(35.0) | 121(30.9) | 48(19.5) | 68(26.8) | 99(29.7) | 145(40.8) | 61(26.1) | 129(52.7) | 60(22.6) | 144(52.6) | 48(30.0) | 87(58.8) |
| 40-59years | 29(31.9) | 36(40.0) | 103(46.4) | 96(43.4) | 40(37.7) | 45(42.9) | 72(41.4) | 89(48.4) | 40(39.6) | 61(58.1) | 43(38.4) | 61(51.7) | 19(29.2) | 34(55.7) |
| ≥60years | 0(0.0) | 3(42.9) | 10(58.8) | 10(55.6) | 3(42.9) | 4(44.4) | 7(58.3) | 5(45.5) | 2(33.3) | 3(50.0) | 2(40.0) | 2(40.0) | 0(0.0) | 1(25.0) |
| **Treatments patterns, n (%)** |  |  |  |  |  |  |  |  |  |  |  |  |  |  |
| Surgeries | 52(36.4) | 66(43.1) | 237(49.0) | 194(41.1) | 74(38.7) | 87(43.7) | 154(45.6) | 190(51.8) | 89(41.4) | 146(64.0) | 87(38.0) | 146(60.3) | 53(39.0) | 69(58.5) |
| Medical | 0(0.0) | 5(21.7) | 3(15.8) | 6(31.6) | 1(7.1) | 1(7.7) | 1(5.6) | 2(11.8) | 1(11.1) | 2(25.0) | 1(7.1) | 2(12.5) | 0(0.0) | 4(36.4) |
| Surgeries and Medical | 17(18.1) | 22(22.9) | 31(27.7) | 37(31.9) | 19(20.7) | 25(27.2) | 26(24.8) | 34(31.2) | 24(29.3) | 37(44.6) | 20(25.3) | 42(50.0) | 19(35.8) | 34(63.0) |
| Surgeries and Radiotherapy | 1(7.7) | 1(9.1) | 3(9.1) | 7(21.2) | 1(4.0) | 5(19.2) | 4(14.8) | 12(42.9) | 1(5.6) | 11(61.1) | 2(7.7) | 8(32.0) | 0(0.0) | 8(72.7) |
| Medical and Radiotherapy | 0(0.0) | 0(0.0) | 1(33.3) | 1(33.3) | 0(0.0) | 0(0.0) | 1(20.0) | 2(40.0) | 0(0.0) | 1(16.7) | 0(0.0) | 2(33.3) | 1(20.0) | 2(50.0) |
| Surgeries, Medical and Radiotherapy | 3(5.3) | 9(15.5) | 5(8.8) | 8(14.5) | 4(5.6) | 12(16.4) | 8(9.9) | 24(28.9) | 4(7.1) | 25(40.3) | 11(14.5) | 35(47.9) | 7(14.6) | 23(50.0) |
| **First treated with surgeries, n (%)** |  |  |  |  |  |  |  |  |  |  |  |  |  |  |
| Microscopic approach | 40(26.5) | 47(30.3) | 189(43.0) | 153(36.2) | 58(27.9) | 68(32.5) | 143(40.1) | 169(45.1) | 87(34.9) | 141(54.4) | 91(35.1) | 150(55.1) | 64(32.5) | 93(55.7) |
| Endoscopic approach | 28(27.5) | 42(39.3) | 80(43.5) | 79(41.4) | 38(31.1) | 47(36.4) | 47(35.1) | 74(49.7) | 29(36.3) | 64(70.3) | 25(27.5) | 59(62.8) | 13(32.5) | 28(75.7) |
| Open operation | 0(0.0) | 0(0.0) | 1(20.0) | 1(16.7) | 2(28.6) | 2(28.6) | 0(0.0) | 1(25.0) | 0(0.0) | 2(50.0) | 1(25.0) | 3(60.0) | 0(0.0) | 3(60.0) |
| **Starting time of follow-up, n (%)** |  |  |  |  |  |  |  |  |  |  |  |  |  |  |
| 2012~2015 | 21(25.0) | 24(28.2) | 81(41.3) | 65(33.7) | 42(32.8) | 40(31.7) | 66(32.8) | 84(40.2) | 50(29.8) | 79(49.4) | 56(30.6) | 96(50.3) | 60(30.2) | 104(56.5) |
| 2016~2019 | 45(21.0) | 66(29.3) | 180(38.5) | 170(37.2) | 47(19.6) | 78(31.0) | 116(35.3) | 158(44.8) | 69(32.5) | 142(58.9) | 64(27.0) | 138(56.6) | 14(34.1) | 27(81.8) |
| 2020~2021 | 7(20.6) | 13(38.2) | 18(40.0) | 18(37.5) | 10(34.5) | 12(42.9) | 11(26.8) | 21(47.7) | - | - | - | - | - | - |
| **Adenoma sizes, n (%)** |  |  |  |  |  |  |  |  |  |  |  |  |  |  |
| Micro-adenoma (<10 mm) | 18(47.4) | 19(51.4) | 50(59.5) | 36(42.9) | 19(51.4) | 21(48.8) | 34(49.3) | 37(48.7) | 16(37.2) | 28(59.6) | 16(36.4) | 27(52.9) | 14(46.7) | 19(70.4) |
| Macro-adenoma (≥10 mm) | 50(21.6) | 65(28.6) | 221(40.2) | 200(37.0) | 79(26.2) | 96(31.7) | 156(34.6) | 210(46.2) | 101(35.2) | 179(58.9) | 100(31.8) | 185(57.1) | 62(32.5) | 104(59.1) |
| **Invasion,** **n (%)** |  |  |  |  |  |  |  |  |  |  |  |  |  |  |
| Yes | 11(11.5) | 14(15.1) | 39(21.1) | 37(20.1) | 16(13.9) | 23(19.8) | 36(22.1) | 54(33.1) | 21(21.9) | 54(51.9) | 24(18.6) | 65(49.6) | 18(21.7) | 31(43.7) |
| No | 52(37.7) | 68(44.4) | 228(54.7) | 191(46.7) | 82(42.1) | 88(44.0) | 149(49.0) | 183(54.8) | 90(44.1) | 135(63.7) | 88(44.2) | 133(62.4) | 58(45.3) | 80(70.0) |
| **Diagnostic delay, n (%)** |  |  |  |  |  |  |  |  |  |  |  |  |  |  |
| <4 year | 24(15.7) | 44(27.8) | 117(35.9) | 112(35.3) | 44(21.7) | 60(29.3) | 77(27.1) | 119(40.1) | 40(22.1) | 95(49.2) | 61(26.6) | 127(54.5) | 32(26.0) | 62(55.9) |
| ≥4 year | 49(27.4) | 59(31.7) | 162(42.4) | 141(37.0) | 55(28.4) | 70(34.8) | 117(40.1) | 146(46.5) | 78(38.0) | 125(59.2) | 60(29.7) | 108(50.5) | 48(33.1) | 78(58.2) |

**Supplementary table2**

**Number and proportion of biochemical remission in acromegaly patients in seven different follow-up time groups according to Chinese diagnostic criteria**

| **Characteristic** | **Less than 3 months**  **(n=318)** | **≥3 and <6 months**  **(n=675)** | **≥6 and ≤12 months**  **(n=386)** | **>1 and ≤2 years**  **(n=581)** | **>2 and ≤3 years**  **(n=386)** | **>3 and ≤5 years**  **(n=436)** | **More than 5 years**  **(n=239)** |
| --- | --- | --- | --- | --- | --- | --- | --- |
| **Sum of remission, n (%)** | 63(19.8) | 205(30.4) | 88(22.8) | 197(33.9) | 162(42.0) | 171(39.2) | 99(41.4) |
| **Sex, n (%)** |  |  |  |  |  |  |  |
| Male | 26(19.0) | 90(30.3) | 29(20.1) | 85(35.6) | 66(39.8) | 63(39.6) | 38(40.0) |
| Female | 37(20.4) | 115(30.4) | 59(24.4) | 112(32.7) | 96(43.6) | 108(39.0) | 61(42.4) |
| **Age of onset, n (%)** |  |  |  |  |  |  |  |
| <20years | 2(7.4) | 21(32.8) | 7(19.4) | 16(28.6) | 18(40.9) | 20(40.8) | 12(37.5) |
| 20-39years | 34(17.1) | 94(24.8) | 41(16.9) | 103(30.7) | 89(38.0) | 96(36.1) | 64(44.4) |
| 40-59years | 25(29.1) | 83(38.8) | 36(36.0) | 74(41.3) | 51(51.0) | 53(45.7) | 22(37.3) |
| ≥60years | 2(33.3) | 7(38.9) | 4(57.1) | 4(36.4) | 2(33.3) | 2(40.0) | 1(25.0) |
| **Treatments patterns, n (%)** |  |  |  |  |  |  |  |
| Surgeries | 46(33.1) | 164(35.7) | 65(34.8) | 151(43.6) | 121(55.3) | 117(49.4) | 57(48.7) |
| Medical | 3(14.3) | 5(26.3) | 1(8.3) | 1(5.9) | 1(12.5) | 1(6.7) | 2(18.2) |
| Surgeries and Medical | 11(12.4) | 25(22.5) | 18(20.0) | 21(20.2) | 24(30.8) | 28(35.0) | 24(46.2) |
| Surgeries and Radiotherapy | 1(9.1) | 7(22.6) | 1(4.3) | 9(32.1) | 6(33.3) | 6(24.0) | 4(40.0) |
| Medical and Radiotherapy | 0(0.0) | 1(33.3) | 0(0.0) | 2(40.0) | 0(0.0) | 0(0.0) | 1(25.0) |
| Surgeries, Medical and Radiotherapy | 2(3.6) | 3(5.8) | 3(4.2) | 13(16.5) | 10(17.9) | 19(26.4) | 11(25.0) |
| **First treated with surgeries, n (%)** |  |  |  |  |  |  |  |
| Microscopic approach | 29(20.3) | 129(31.0) | 51(24.9) | 134(37.4) | 108(43.9) | 116(43.8) | 71(43.6) |
| Endoscopic approach | 26(26.5) | 63(35.2) | 28(23.9) | 52(36.9) | 49(57.6) | 42(45.7) | 19(52.8) |
| Open operation | 0(0.0) | 1(20.0) | 2(28.1) | 1(25.0) | 2(50.0) | 3(60.0) | 1(25.0) |
| **Starting time of follow-up, n (%)** |  |  |  |  |  |  |  |
| 2012~2015 | 17(21.0) | 54(28.9) | 28(23.0) | 62(31.3) | 56(37.3) | 74(40.0) | 75(41.9) |
| 2016~2019 | 40(19.6) | 136(30.8) | 52(21.9) | 118(35.2) | 106(45.9) | 97(40.4) | 18(56.3) |
| 2020~2021 | 6(18.2) | 15(32.6) | 8(29.6) | 16(37.2) | - | - | - |
| **Adenoma sizes, n (%)** |  |  |  |  |  |  |  |
| Micro-adenoma (<10 mm) | 15(42.9) | 31(38.8) | 16(43.2) | 33(47.1) | 22(50.0) | 19(39.6) | 16(53.9) |
| Macro-adenoma (≥10 mm) | 39(17.6) | 164(31.2) | 66(22.5) | 155(35.6) | 134(46.5) | 140(44.0) | 75(43.9) |
| **Invasion,** **n (%)** |  |  |  |  |  |  |  |
| Yes | 9(10.1) | 29(16.3) | 16(14.2) | 43(27.0) | 38(38.4) | 45(34.6) | 23(33.3) |
| No | 43(31.9) | 163(41.2) | 65(34.6) | 139(44.1) | 109(54.2) | 106(51.5) | 65(55.1) |
| **Diagnostic delay, n (%)** |  |  |  |  |  |  |  |
| <4 year | 21(14.2) | 89(29.0) | 40(20.3) | 85(30.0) | 69(38.3) | 93(40.4) | 45(41.3) |
| ≥4 year | 42(24.7) | 116(31.5) | 48(25.4) | 112(37.6) | 91(44.6) | 78(37.9) | 54(41.5) |

**Supplementary table3**

**Number and proportion of GH remission, IGF-1 remission in acromegaly patients in seven different follow-up time groups according to Chinese diagnosis criteria**

| **Characteristic** | **Remission of two models per biochemical examination period** | | | | | | | | | | | | | |
| --- | --- | --- | --- | --- | --- | --- | --- | --- | --- | --- | --- | --- | --- | --- |
|  | **Less than 3 months** | | **≥3 and <6 months** | | **≥6 and ≤12 months** | | **>1 and ≤2 years** | | **>2 and ≤3 years** | | **>3 and ≤5 years** | | **More than 5 years** | |
|  | **GH**  **(n=333)** | **IGF-1**  **(n=344)** | **GH**  **(n=715)** | **IGF-1 (n=698)** | **GH**  **(n=403)** | **IGF-1 (n=406)** | **GH**  **(n=603)** | **IGF-1**  **(n=611)** | **GH**  **(n=431)** | **IGF-1**  **(n=406)** | **GH**  **(n=475)** | **IGF-1**  **(n=447)** | **GH**  **(n=304)** | **IGF-1**  **(n=245)** |
| **Sum of remission, n (%)** | 125(37.5) | 103(29.9) | 402(56.2) | 253(36.2) | 155(38.5) | 130(32.0) | 315(52.2) | 265(43.4) | 243(56.4) | 222(54.7) | 251(52.8) | 235(52.6) | 160(52.6) | 140(57.1) |
| **Sex, n (%)** |  |  |  |  |  |  |  |  |  |  |  |  |  |  |
| Male | 55(39.3) | 40(26.7) | 194(62.2) | 111(35.5) | 61(39.6) | 41(26.3) | 146(58.4) | 116(44.8) | 104(57.8) | 92(51.7) | 90(52.0) | 83(49.7) | 61(47.7) | 56(57.1) |
| Female | 70(36.3) | 63(32.5) | 208(51.6) | 142(36.9) | 94(37.8) | 89(35.6) | 169(47.9) | 149(42.3) | 139(55.5) | 130(57.0) | 161(53.3) | 152(54.3) | 99(56.3) | 84(57.1) |
| **Age of onset, n (%)** |  |  |  |  |  |  |  |  |  |  |  |  |  |  |
| <20years | 6(20.7) | 6(19.4) | 34(50.0) | 26(38.2) | 10(26.3) | 13(34.2) | 26(45.6) | 26(42.6) | 26(55.3) | 27(56.3) | 25(49.0) | 28(56.0) | 19(45.2) | 18(56.3) |
| 20-39years | 69(33.5) | 58(26.9) | 201(49.8) | 121(30.9) | 81(32.1) | 68(26.8) | 165(47.4) | 145(40.8) | 135(52.1) | 129(52.7) | 144(49.1) | 144(52.6) | 95(51.6) | 87(58.8) |
| 40-59years | 48(52.2) | 36(40.0) | 155(68.9) | 96(43.4) | 60(56.6) | 45(42.9) | 117(62.9) | 89(48.8) | 76(66.1) | 61(58.1) | 79(62.7) | 61(51.7) | 43(58.1) | 34(55.7) |
| ≥60years | 2(33.3) | 3(42.9) | 12(66.7) | 10(55.6) | 4(57.1) | 4(44.4) | 7(58.3) | 5(45.5) | 4(50.0) | 3(50.0) | 3(60.0) | 2(40.0) | 3(75.0) | 1(25.0) |
| **Treatments patterns, n (%)** |  |  |  |  |  |  |  |  |  |  |  |  |  |  |
| Surgeries | 77(53.5) | 66(43.1) | 323(65.9) | 194(41.1) | 109(55.3) | 87(43.7) | 239(65.8) | 190(51.8) | 173(68.9) | 146(64.0) | 169(64.0) | 146(60.3) | 95(60.5) | 69(58.5) |
| Medical | 5(22.7) | 5(21.7) | 7(36.8) | 6(31.6) | 2(14.3) | 1(7.7) | 1(5.6) | 2(11.8) | 1(11.1) | 2(25.0) | 5(33.3) | 2(12.5) | 3(27.3) | 4(36.4) |
| Surgeries and Medical | 31(33.0) | 22(22.9) | 52(46.0) | 37(31.9) | 36(39.1) | 25(27.2) | 41(38.7) | 34(31.2) | 40(47.1) | 37(44.6) | 42(50.6) | 42(50.0) | 32(54.2) | 34(63.0) |
| Surgeries and Radiotherapy | 4(30.8) | 1(9.1) | 11(33.3) | 7(21.2) | 1(4.0) | 5(19.2) | 11(39.3) | 12(42.9) | 11(52.4) | 11(61.1) | 9(32.1) | 8(32.0) | 8(40.0) | 8(72.7) |
| Medical and Radiotherapy | 2(66.7) | 0(0.0) | 1(33.3) | 1(33.3) | 0(0.0) | 0(0.0) | 3(60.0) | 2(40.0) | 1(16.7) | 1(16.7) | 1(16.7) | 2(33.3) | 3(60.0) | 2(50.0) |
| Surgeries, Medical and Radiotherapy | 6(10.5) | 9(15.5) | 8(14.0) | 8(14.5) | 7(9.9) | 12(16.4) | 20(24.7) | 24(28.9) | 16(27.6) | 25(40.3) | 24(31.2) | 35(47.9) | 18(35.3) | 23(50.0) |
| **First treated with surgeries, n (%)** |  |  |  |  |  |  |  |  |  |  |  |  |  |  |
| Microscopic approach | 62(40.8) | 47(30.3) | 267(59.9) | 153(36.2) | 87(41.2) | 68(32.5) | 218(58.3) | 169(45.1) | 172(61.6) | 141(54.4) | 171(59.4) | 150(55.1) | 117(56.0) | 93(55.7) |
| Endoscopic approach | 44(43.1) | 42(39.3) | 115(62.2) | 79(41.4) | 57(46.0) | 47(36.4) | 82(56.9) | 74(49.7) | 59(64.1) | 64(70.3) | 55(54.5) | 59(62.8) | 27(57.4) | 28(75.7) |
| Open operation | 0(0.0) | 0(0.0) | 1(20.0) | 1(16.7) | 2(28.6) | 2(28.6) | 2(50.0) | 1(25.0) | 2(50.0) | 2(50.0) | 3(60.0) | 3(60.0) | 1(20.0) | 3(60.0) |
| **Starting time of follow-up, n (%)** |  |  |  |  |  |  |  |  |  |  |  |  |  |  |
| 2012~2015 | 36(42.9) | 24(28.2) | 116(58.9) | 65(33.7) | 56(43.8) | 40(31.7) | 105(51.7) | 84(40.2) | 94(55.3) | 79(49.4) | 109(56.5) | 96(50.3) | 126(55.0) | 104(56.5) |
| 2016~2019 | 75(34.9) | 66(29.3) | 256(54.4) | 170(37.2) | 86(35.1) | 78(31.0) | 183(52.6) | 158(44.8) | 149(58.7) | 142(58.9) | 140(51.7) | 138(56.6) | 24(52.2) | 27(81.8) |
| 2020~2021 | 14(41.2) | 13(38.2) | 30(63.8) | 18(37.5) | 13(43.3) | 12(42.9) | 26(55.3) | 21(47.7) | - | - | - | - | - | - |
| **Adenoma sizes, n (%)** |  |  |  |  |  |  |  |  |  |  |  |  |  |  |
| Micro-adenoma (<10 mm) | 25(65.8) | 19(51.4) | 68(80.0) | 36(42.9) | 26(70.3) | 21(48.8) | 47(63.5) | 37(48.7) | 35(70.0) | 28(59.6) | 35(68.6) | 27(52.9) | 20(66.7) | 19(70.4) |
| Macro-adenoma (≥10 mm) | 84(36.2) | 65(28.6) | 317(57.0) | 200(37.0) | 120(39.1) | 96(31.7) | 253(56.1) | 210(46.2) | 194(60.2) | 179(58.9) | 194(55.7) | 185(57.1) | 125(55.6) | 104(59.1) |
| **Invasion,** **n (%)** |  |  |  |  |  |  |  |  |  |  |  |  |  |  |
| Yes | 23(24.0) | 14(15.1) | 71(37.8) | 37(20.1) | 27(23.5) | 23(19.8) | 69(41.1) | 54(33.1) | 49(45.8) | 54(51.9) | 63(44.7) | 65(49.6) | 43(46.7) | 31(43.7) |
| No | 76(54.7) | 68(44.4) | 306(72.7) | 191(46.7) | 118(59.0) | 88(44.0) | 222(68.1) | 183(54.8) | 167(72.0) | 135(63.7) | 153(68.0) | 133(62.4) | 98(64.5) | 80(70.0) |
| **Diagnostic delay, n (%)** |  |  |  |  |  |  |  |  |  |  |  |  |  |  |
| <4 year | 43(28.1) | 44(27.8) | 170(52.0) | 112(35.3) | 73(35.4) | 60(29.3) | 137(46.4) | 119(40.1) | 109(53.7) | 95(49.2) | 126(51.0) | 127(54.5) | 76(53.5) | 62(55.9) |
| ≥4 year | 82(45.6) | 59(31.7) | 232(59.8) | 141(37.0) | 82(41.6) | 70(34.8) | 178(57.8) | 146(46.5) | 132(58.4) | 125(59.2) | 125(54.8) | 108(50.5) | 84(51.9) | 78(58.2) |

**Supplementary figure1**

**Total number and sex difference in different age groups of diagnosis**

**
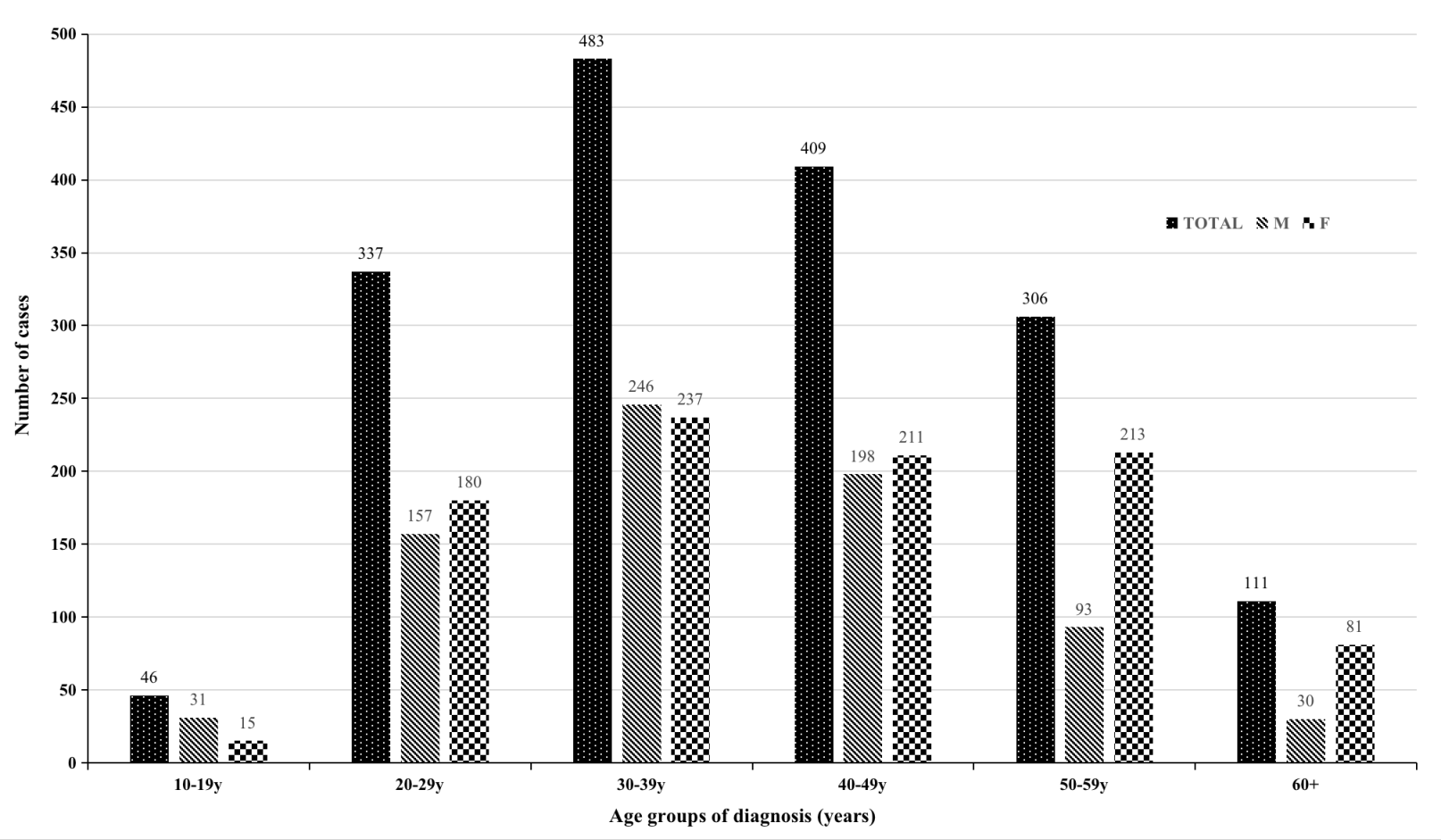
**
